# Comprehensive Collection and Prediction of ABC Transmembrane Protein Structures in the AI Era of Structural Biology

**DOI:** 10.3390/ijms23168877

**Published:** 2022-08-09

**Authors:** Hedvig Tordai, Erzsebet Suhajda, Ian Sillitoe, Sreenath Nair, Mihaly Varadi, Tamas Hegedus

**Affiliations:** 1 Department of Biophysics and Radiation Biology, Semmelweis University, 1085 Budapest, Hungary; 2 Faculty of Electrical Engineering and Informatics, Budapest University of Technology and Economics, 1111 Budapest, Hungary; 3 Wigner Research Centre for Physics, 1121 Budapest, Hungary; 4 Department of Structural and Molecular Biology, University College London, London WC1E 6BT, UK; 5 European Bioinformatics Institute, European Molecular Biology Laboratory, Hinxton CB10 1SD, UK; 6 ELKH-SE Biophysical Virology Research Group, Eötvös Loránd Research Network, 1052 Budapest, Hungary

**Keywords:** protein structure, protein complex, ABC transporters, AlphaFold2, AF-multimer, structure database, 3D-Beacons

## Abstract

The number of unique transmembrane (TM) protein structures doubled in the last four years, which can be attributed to the revolution of cryo-electron microscopy. In addition, AlphaFold2 (AF2) also provided a large number of predicted structures with high quality. However, if a specific protein family is the subject of a study, collecting the structures of the family members is highly challenging in spite of existing general and protein domain-specific databases. Here, we demonstrate this and assess the applicability and usability of automatic collection and presentation of protein structures via the ABC protein superfamily. Our pipeline identifies and classifies transmembrane ABC protein structures using the PFAM search and also aims to determine their conformational states based on special geometric measures, conftors. Since the AlphaFold database contains structure predictions only for single polypeptide chains, we performed AF2-Multimer predictions for human ABC half transporters functioning as dimers. Our AF2 predictions warn of possibly ambiguous interpretation of some biochemical data regarding interaction partners and call for further experiments and experimental structure determination. We made our predicted ABC protein structures available through a web application, and we joined the 3D-Beacons Network to reach the broader scientific community through platforms such as PDBe-KB.

## 1. Introduction

ABC (ATP Binding Cassette) proteins play crucial roles in diverse biological functions from bacteria to man [1,2]. Importantly, human ABC transmembrane proteins are involved in several pathophysiological processes [1,3,4]. Mutations can affect their folding, assembly, trafficking, plasma membrane stability, and function, leading to decreased functional expression [5,6]. Therefore, understanding the effect of mutations on their structure and dynamics is highly important. Several family members extrude xenobiotics (e.g., toxic molecules and therapeutic drugs) from the cell, thus, learning the substrate recognition mechanism of these multidrug transporters can improve drug development and may prevent unwanted drug interactions [2,7]. Consequently, determining the atomic level 3D structure of ABC proteins is a widely researched area [8,9,10,11,12]. For example, various steps of structure-based drug design, such as selecting the appropriate protein target, understanding its pathological mechanism at the molecular level, and developing a new therapeutic substance or redesigning a drug require the knowledge of the target protein structure at the atomic level [13].

The major protein structure determination method was X-ray crystallography for several decades [14]. This technique requires vast resources, especially in the case of transmembrane proteins, which are the target of a large fraction of prescription drugs [15,16]. The examined protein needs to be isolated and purified in a large quantity and the process of crystallization requires a trial-and-error approach [17]. Furthermore, it is rare to obtain structural information on the membrane environment from the crystal. In recent years, cryo-electron microscopy (cryo-EM) emerged and became the ultimate structure determination method for transmembrane proteins [18]. The cryo-EM maps may also include information about the membrane environment [19]. Despite these recent advances in the structure determination of transmembrane proteins, they only make up ~5% of all the protein structures in the Protein Data Bank, while close to 50% of prescription drugs target transmembrane proteins [15,16].

Researchers utilized enormous resources in the last decades to predict protein structures from sequence information [20]. In recent years, an algorithm based on deep learning, AlphaFold2, emerged with remarkable accuracy in this task [21]. Its high quality and speed of structure prediction resulted in a vast database of predicted structures, the AlphaFold Protein Structure Database, which currently holds close to 1,000,000 protein structures [22]. AlphaFold2 (AF2) and AlphaFold-Multimer are serving now as ultimate tools for learning protein structures that would be hard to resolve experimentally, such as protein complexes and transmembrane (TM) proteins. Although AF2 was not specifically trained to predict transmembrane proteins, we have recently demonstrated on ABC proteins that this novel deep learning method is able to provide high-quality structures of transmembrane proteins [23]. RoseTTAFold [24] and trRosetta [25] have also been published as high-accuracy structure predictors, based on various deep learning approaches.

**Figure 1:**
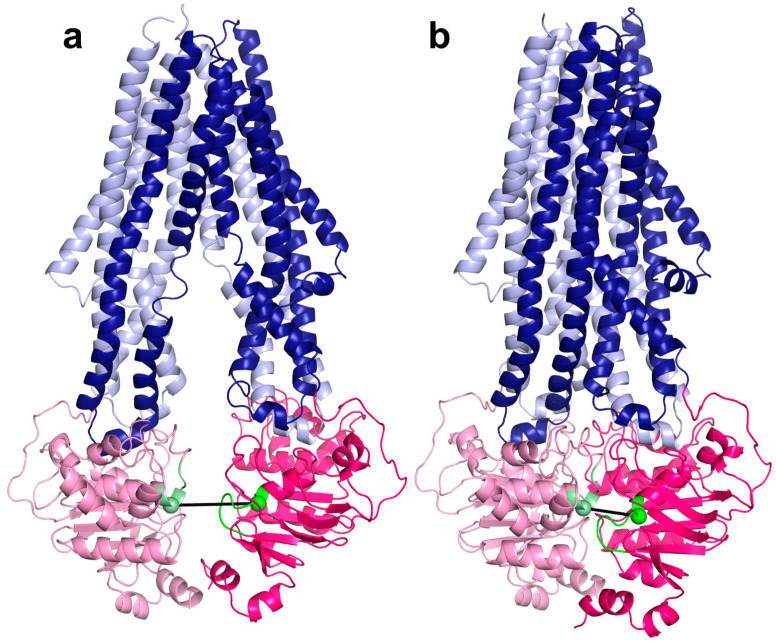
Inward-facing ((**a**), PDB ID: 7a69) and inward-closed ((**b**), PDB ID: 6c0v) structures of ABCB1/MDR1/Pgp. Light and dark blue colors: TM domains; pink and hot pink: NBDs; green and pale green: the last residue in the Walker A and ABC signature helices, respectively; black: the conftor/distance between the selected amino acids from Walker A and signature.

ABC proteins are an excellent group of proteins to study AF2 performance on TM proteins [23] since their TM domains are not conserved because of their heterogeneous transport functions. Therefore, the structures of their transmembrane domains exhibit various scaffolds that currently enable transmembrane ABC structures to be clustered into nine distinct structural classes (Figure S1) [26]. In contrast, the nucleotide-binding domains (NBDs) of ABC proteins are highly conserved and contain Walker sequences and the ABC signature or fingerprint [8]. Notably, an ATP binding site is formed by the Walker A and B from one NBD and signature from a second NBD. Therefore, the functional form of a TM ABC protein includes two NBDs and TMDs. These domains can be encoded in one polypeptide chain in the case of full transporters, in two chains in the case of half transporters working in dimeric forms, or in four chains [27,28]. The structures of ABC proteins can be observed in various conformations [8,29]. For example, proteins with Pgp-like structures (Figure 1) exhibit a widely open conformation towards the intracellular space, capable of substrate binding, in the absence of ATP. When two ATP molecules are bound, the NBDs close and form a tight interaction, and the extracellular ends of TM helices open to provide a dissociation site towards the extracellular space. These conformations are associated with the steps of the alternating access mechanism [9,30]. Other types of proposed mechanisms for ABC proteins include the peristaltic or credit card swipe mechanisms. For transporters operating with these mechanisms, the aforementioned two distinct conformations cannot be distinguished so promptly [8,31,32].

Although various structures of numerous ABC proteins were determined in the last years using cryo-electron microscopy, 25 of the 44 physiologically and pathologically important human ABC transmembrane proteins lack experimental structures in the PDB. The AlphaFold DB, co-developed by EMBL-EBI and DeepMind [22], supplemented the set of resolved transporters, albeit only full-length transporters, since only single-chain structures are available from this database. While the significant increase in the number of reliable structures for transmembrane proteins is a welcome change, the fact that the structures have to be collected from multiple data providers poses a potential challenge to performing comparative computational and experimental studies. Although both PDB and AF2 structures can be searched with a structure for homologous ones using the DALI server [33], it cannot reliably distinguish different ABC TM folds (https://abc3d.hegelab.org/dali.html). A different approach to solve this issue is the use of the SCOPe domain database [34], but it contains only three types of ABC folds (f.22: ABC transporter involved in vitamin B12 uptake, f.35: Multidrug efflux transporter AcrB transmembrane domain, and f.37: ABC transporter transmembrane region). To tackle the challenges associated with ABC protein structure collection, we built an automated pipeline that collects ABC protein structures from both the PDB and AlphaFold DB [22,35]. We supplemented the structures with relevant metadata (e.g., conformational state, structural family, method of resolving structure) and made them available through a web application (https://abc3d.hegelab.org). We also predicted the functional unit structures of human half transporter dimers using AlphaFold-Multimer [36]. In order to make our data set more accessible, we also linked our service (https://3dbeacon.hegelab.org) to the 3D-Beacons Network (https://3d-beacons.org).

## 2. Results

### 2.1. Connections between Transmembrane ABC Proteins Structures and Pfam Profiles

**Figure 2:**
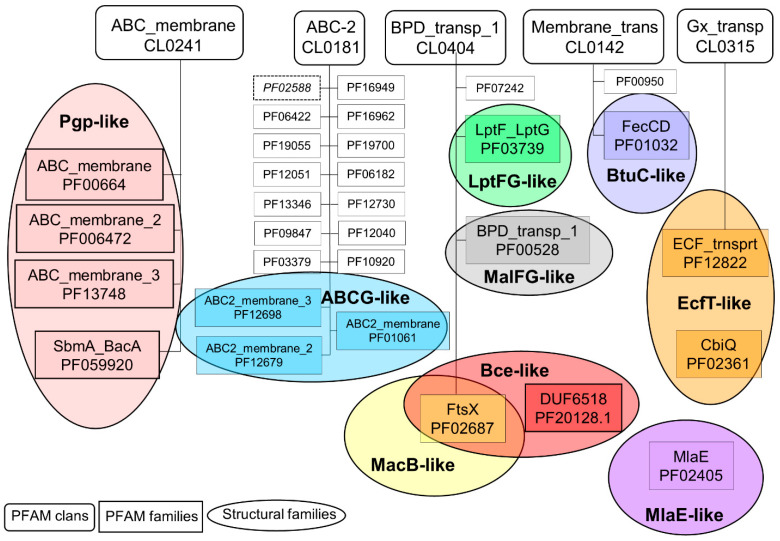
Pfam and structural classes of ABC proteins. Pfam clans (rounded boxes) with ABC protein hits are depicted. Those Pfam families (boxes), which include proteins with experimental structures, are circled, colored, and labeled in bold. The labels were selected based on a widely known member of the structural family (e.g., Pgp-like). PF02588/YitT_membrane is in italic and with a dotted outline since it likely does not involve ABC family members.

Running our pipeline yielded 325 PDB structures (as of 29 December 2021). We selected the top-scoring TMD matches of our Pfam searches as the base for classification (Figure 2). We found only three incorrect biological unit files (3wme, 3wmf, and 3wmg), which contained only half of the functional unit of an ABC transporter (1 NBD and 1 TMD). Instead of numbered structural classes [26], we use names related to the most known representative member of a class (e.g., Pgp-like and BtuCD-like) (Figure S1), since we find this not only more informative than numbers, but the recently renumbered classes cause confusions in publications of the last decades (e.g., type I ABC exporter in the old system corresponds to type IV transporter structure in the recently proposed system [26]). In addition, the numbering strongly suggests an order of the structural families (e.g., an ordering based on the evolutionary level of TM domains) but that is not the case.

The investigation of sequence and structure conservation led to important and interesting information regarding this diverse protein family. The HMM search showed matches in five Pfam clans (Figure 2). The *ABC_membrane* clan includes four PFAM families, exhibiting Pgp-like structures. Of these four families, most of the existing experimental structures belong to the *ABC_membrane* family. The *ABC_membrane2* family contains ABCD [37,38] and a mycobacterial ABC transporter, Rv1819c [39] structures, while no experimental structure is associated with the *ABC_membrane3* family. However, trRosetta and AlphaFold2 predictions also suggested a Pgp-like architecture for members of this family (e.g., https://alphafold.ebi.ac.uk/entry/Q5F7D3 accessed on 20 June 2022). Importantly, we found that automatic computational models need careful interpretation. The trRosetta prediction of a protein of this Pfam family (UniProt ACC: Q9CNM3) did not contain the full sequence. In addition, neither the trRosetta nor the AlphaFold prediction took the dimeric nature of this protein into account (Figure S2). Interestingly, a member of the fourth family (*SbmA_BacA*) was demonstrated to form a homodimer of two TMDs and to function without any NBDs, thus it cannot be considered a real ABC protein (PDB ID: 7p34) [40].

The *ABC-2* clan includes proteins that exhibit, or potentially exhibit ABCG2-like structures. Most of the related experimental structures belong to the *ABC2_membrane* and *ABC2_membrane_3* families including ABCG and ABCA proteins, respectively. The *ABC2_membrane* Pfam entry matches the first five TM helices of ABCG proteins. The exclusion of TM6 is most likely caused by the non-conserved extracellular loop between TM5 and TM6. TM1 was also not matched in most ABCA TMD by the *ABC2_membrane_3* profile, which involves a portion of their large extracellular loop (Figure S3). Experimental ABCG2-like structures (6xjh and 6xji) in the *ABC2_membrane_2* family were determined for the same protein, PmtCD, exporting bacterial toxins [40]. The remaining families involve trRosetta or AlphaFold2 computational models. Most of the sequences of these proteins incorporate transmembrane domains and some other domains (e.g., Sodium/calcium exchanger, AAA, Dihydrodipicolinate synthetase, and Phosphotransferase enzyme in the CcmB and DUF3533 Pfam families), which are not characteristic for ABC proteins. These proteins may or may not function without NBDs and are good targets for studying the evolution of ABC proteins. In contrast to other *ABC-2* clan members, the Pfam profile of ABC2_membrane_7, PDR_CDR, and YitT_membrane (PDB ID: 3hlu) does not match membrane domains, but soluble regions instead. In addition, the AF2-predicted TMD of YitT (e.g., https://alphafold.ebi.ac.uk/entry/O34792, accessed on 20 June 2022) does not resemble an ABCG2-like structure, thus this family likely does not belong to this group of proteins [23].

The *BPD_transp_1* clan includes four families with diverse structures (Figure 2 and Figure S1). Three Pfam families include proteins with experimental structures belonging to different structural classes (MacB-, Bce-, LptFG-, and MalFG-like). Notably, the known Bce-like structures (PDB IDs: 7tcg and 7tch [41]) involve two transmembrane domains with MacB-like and Bce-like folds on a single polypeptide chain. The fourth family, DUF1430, is defined by a soluble domain. In these proteins, cytosolic domains likely prohibit the binding of NBDs to the TMDs, thus they may not be considered real ABC proteins.

Members of both the *ECF_trnsprt* family (*Gx_transp* clan) and the *CbiQ family* (no parent clan) exhibit an EcfT-like structure. The *FecCD* and ABC-3 families in the *Membrane_trans* clan possess BtuCD-like structures.

### 2.2. Conformational States of ABC Protein Structures

Two major conformational states of ABC proteins, called inward-facing and outward-facing, can be distinguished [9,30]. Although this categorization is slightly simplified, since a lot of inward-facing transporters are in an occluded or some intermediate state, we aimed to group the structures focusing on these two states for the sake of automation. Since the outward-facing state is related to the ATP-induced association of the two NBDs, theoretically we could divide the structures into these two conformational states based on the presence or absence of nucleotides in the structure. However, there are several structures with bound nucleotides (e.g., 6z5u, 7oj8, 7ojh, and 7oz1), exhibiting separated NBDs (Figure S4). For example, in the 6z5u structure, the non-hydrolyzable ATP analogue, AppNHpANP, was not sufficient to trigger the transition from bottom-open to bottom-closed state [42]. As another example, a recent ABCG2 structure was determined under turnover conditions and the presence of ATP did not drive a complete closure of the two NBDs (Figure S4) [43].

Since the presence or absence of nucleotides proved to be an inefficient way of categorizing structures, we then tried to do so by calculating the RMSD between each individual structure and two typical reference structures representing either inward- or outward-facing conformations of the structural family the protein belongs to (Table S1). All the structures and their labels were manually validated after classification. We found a higher number of mislabeled structures only in the Pgp-like family, where 18 out of 118 inward-facing structures were classified as outward-facing. These RMSD calculations also uncovered some flaws in currently used structural alignment tools demonstrated in Figure S5.

**Figure 3:**
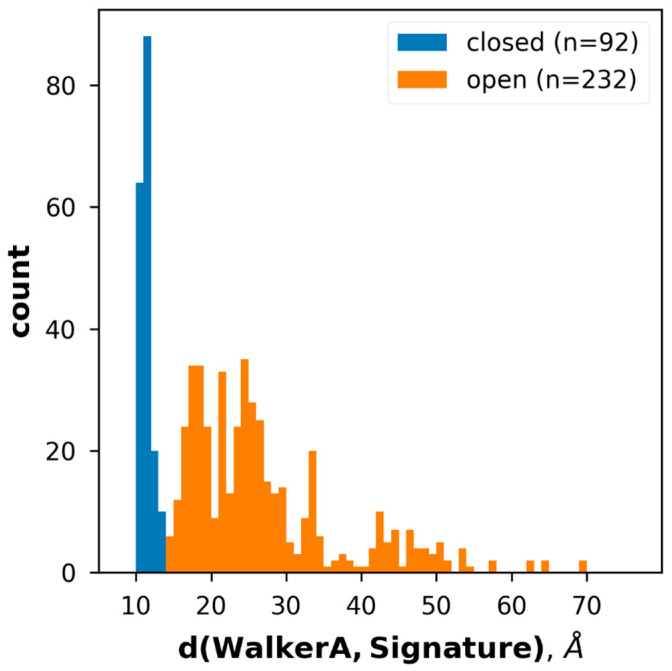
Grouping experimental structures based on the length of conftor(WA/SIG). The distribution of distance values of all collected ABC protein structures.

We have developed 3D vector-based measures, conftors, to describe the relative orientation of domains and highlight structural differences in ABC protein structures [44]. Since the inward- and outward-facing conformation correlates with the level of NBD association (Figure 1 and Figure S1), we defined |conftor(WA/SIG)|, the distance between the Walker A in one of the NBDs and the signature motif from the other NBD to classify the conformations (Figure 1). For this definition, two residues from the Walker A (GXXXXG**K**T) and the signature (LS**G**GQ) sequences were selected based on their spatial location. Since these Lys and Gly are located at helix ends and not in loops, noisy fluctuations in the distance between their Cα atoms were prevented. These positions were identified by aligning the query NBDs to a reference NBD (Pgp NBD1 from 6c0v) using the TM-align algorithm and not by simple sequence search, since there are many ABC proteins with degenerate, non-conserved ATP-binding sites (e.g., CFTR/ABCC7 possesses a degenerate signature motif [45,46]). The distribution of the distances between Walker A and signature calculated for all structures (Figure 3) indicated a cutoff value of 14 Å for distinguishing bottom-closed and bottom-open conformations. Indeed, visual inspection of structures with|conftor(WA/SIG)| = 14 ± 2 Å confirmed this value as a rational cutoff. We aimed to identify the source of very large NBD separation, thus we compared the distance values of X-ray and cryo-EM structures. Large NBD distances were observed in structures of both datasets (Figure S6).

In spite of the above limitations, we found this type of structural classification of inward-facing and outward-facing categories important, since the opening level and the dynamic alterations in the opening affect the substrate access to the central binding pocket [47]. This is clearly evident in the case of some Pgp-like structures, which exhibit an ATP-bound conformation with a well-defined, large, outward open cavity (Figure S7) [48]. In contrast, the difference in the TMDs of ABCG structures is subtle between the apo and ATP-bound states (Figure S7). Although the TM helices are reorganized upon ATP binding, no large opening on their extracellular end is observed. It is also important to note that the static ABC structures cover different parts of a continuous space of possible conformations. This is highly prominent in the case of inward-facing conformations, which exhibit a wide range of opening levels (between 14 and 86 Å) and also various conformational states of the two NBDs relative to each other. We highlight two examples associated with this issue. First, ABCG2 (PDB ID: 6vxf) has an additional conformation named inward-facing-closed [49]. In this ATP-bound conformation, the NBDs are separated, but the intracellular ends of some TM helices are somewhat more closed than in the inward-facing conformation (Figure S7). Although the difference between TM helix ends is subtle, it makes the large drug binding cavity in the TM region inaccessible from the intracellular space. However, since the overall conformation is almost identical to the inward-facing ABCG2 structures (RMSD = 1.736 Å) we classified this structure as inward-facing. Second, the NBDs in ECF proteins show a closed conformation both in the ATP-bound (5d3m) and apo structures (4huq, 4rfs, 4hzu, 5jsz, 5x3x, 5x41, 6fnp, 6zg3, 7nnt, and 7nnu), which is most likely caused by their special TMDs and an unconventional transport mechanism [50,51,52,53]. In other types of ABC transporters, each TM domain possesses at least one coupling helix, responsible for transmitting the conformational changes from the NBDs to TMDs. In ECF transporters, the EcfT TM domain provides two long coupling helices, contacting with the two NBD/ATPase domains and stabilizing the complex [52]. This structural feature contributes to keeping the NBDs and EcfT in close proximity to each other during the dissociation of the substrate binding EcfS TM domain and probably prohibits a large separation of NBDs even in the absence of ATP. Most of the WA/SIG distance values (19 out of 2 × 11) of ECF transporters are between 14 and 19 Å and three are slightly below 14 Å.

### 2.3. ABC Protein Structures Predicted by AlphaFold2

**Figure 4:**
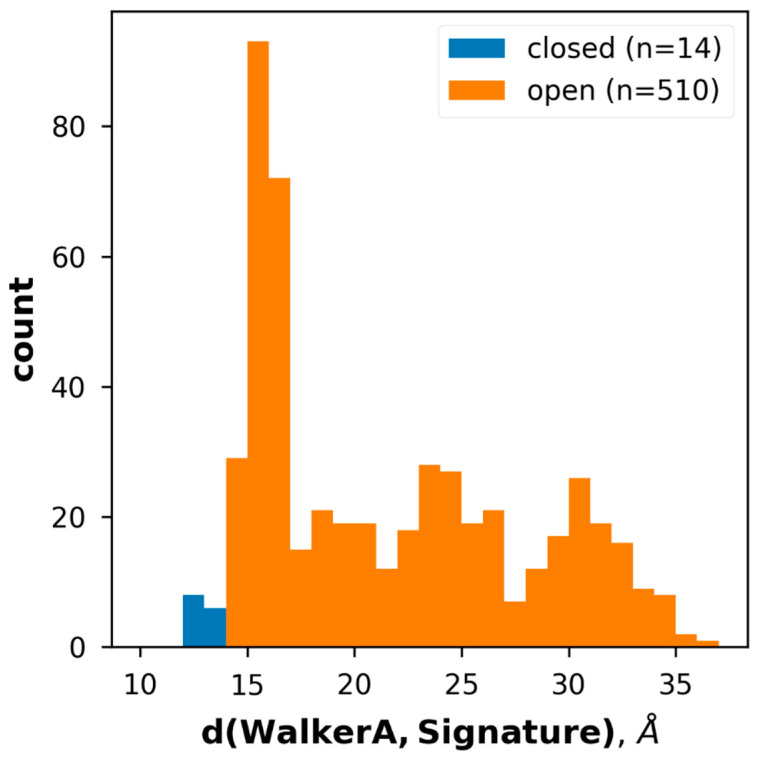
Grouping AF2 structures based on the length of conftor(WA/SIG). The distribution of distance values of all collected AF2-predicted, full ABC structures.

The AlphaFold Protein Structure Database contains predicted structures for most of the protein sequences in the UniProt database. We collected ABC protein structures from this AF database following the same procedure as in the case of the experimentally determined structures from the PDB, namely by using Pfam searches. However, since the AlphaFold DB only contains monomeric structures, we gathered only full transporters, which encoded two NBDs and two TMDs within a single polypeptide chain. As a result, the hits belonged only to Pgp-like and ABCG2-like structural classes. While the open structures were overrepresented in the AF-prediction of 21 proteomes (Figure 4), the numbers of open/closed conformations are more balanced in the prediction of the SwissProt dataset (101 vs. 414 and 163 vs. 136 in the Pgp-like and ABCG2-like families). It is important to note that the bottom-closed, ATP-bound conformation may represent a short-lived state, resulting in a lower number of ATP-bound structures in the experimental dataset. Since these experimental structures served as the AF training set, they possibly biased the AF-predictions toward bottom-open conformations.

We noticed that there is no available experimental or AlphaFold-predicted structural data for some human dimeric ABC proteins. Since we found it critical to generate the functional forms of human ABC protein structures that can facilitate both learning their structure/function relationship and drug development, we predicted dimeric human ABC structures. Previously, we have shown that AlphaFold2 provided high-quality structures for both homodimeric and heterodimeric ABC proteins [23]. However, AlphaFold-Multimer has recently been released, and it has a significantly better performance on heterodimeric complexes when compared to the original AF2 [36]. Using AF-Multimer, we performed test multimer runs for ABC dimers with known structures (e.g., ABCG5/ABCG8, PDB IDs: 5do7, 7jr7, 7r87-7r8b) that yielded high-quality structures (Table S2). Then we performed predictions for proteins with no available structures in the PDB or which had longer unresolved regions (Table S2). For example, several ABCB family members possess an additional N-terminal TM domain (TMD0) which is not resolved or exhibits low resolution (Figure S8). Interestingly, in our AlphaFold predictions, there were no contacts between the core and TMD0, but the additional TMD0 helices were rationally oriented according to a putative membrane bilayer.

**Figure 5:**
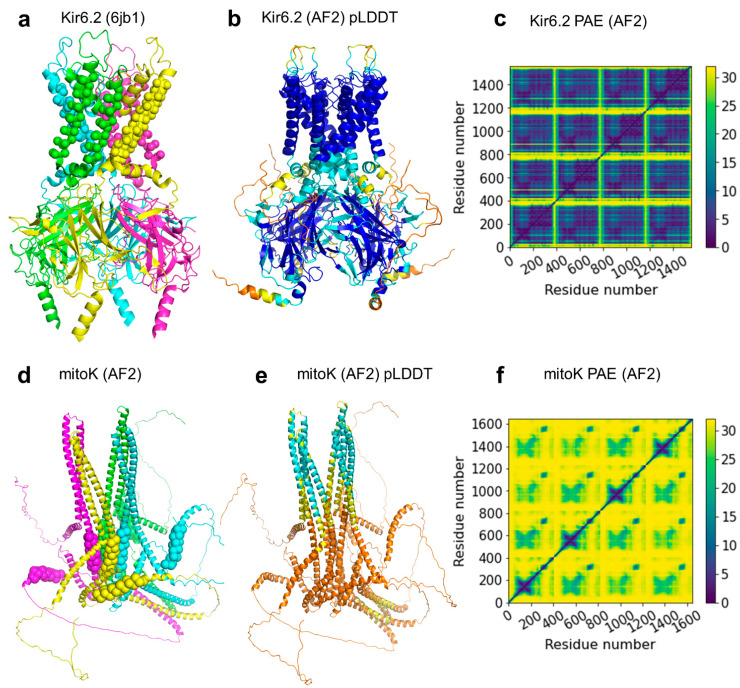
AF2 prediction of the mitoK tetramer. (**a**) The central tetramer of the Kir6.2 potassium channel from the octamer structure with SUR1 (PDB ID: 6jb1). The four chains are colored differently and spheres indicate transmembrane regions. (**b**) The Kir6.2 tetramer predicted by AF-Multimer exhibit high pLDDT scores (blue and turquoise regions). The RMSD calculated for TMDs was 0.8 Å when compared to its experimental structure. (**c**) The plot of predicted align errors (PAE) indicates low values, thus a predicted structure with high quality. (**d**) The AF-predicted structure of mitoK, colored by chains, does not resemble a potassium channel architecture. Spheres indicate transmembrane regions. (**e**) The yellow and orange colors of the same structures correspond to poorly predicted regions with low pLDDT scores. (**f**) The high values of the PAE plot also indicate unreliable structure prediction for the mitoK tetramer.

One of the most exciting tasks was to test if AF-Multimer was able to build the mitoSUR complex. Sulfonylurea receptors (SUR1/ABCC8 and SUR2/ABCC9) are octamers of four SURs and four inward-rectifying potassium Kir channels (Figure 5) [54,55]. Similar to ABCC8 and ABCC9, ABCB8 was found to regulate potassium current [56]. ABCB8 functions in the inner membrane of mitochondria and mitoK (alternative name: Coiled-coil domain-containing protein 51, CCDC51) were identified as its potassium channel partner [56]. In order to build the complex structure, we submitted eight copies of the half transporter ABCB8 and four copies of mitoK sequences to AF-Multimer. No reasonable structures were built. We also predicted the structure of four mitoK proteins without ABCB8. A complex was built, in which the pore was delineated by coiled-coil regions and the putative transmembrane helices were not aligned to match a lipid bilayer (Figure 5). Thus, the overall structure did not meet expectations of what a potassium channel should look like. Known potassium channels, even with low sequence similarity, exhibit two TM helices and a reentrant loop between these TM regions, of which the loop provides the selectivity filter with a characteristic amino acid pattern (TMxTVGYG) [57,58]. We could not find any similar potassium selectivity pattern in mitoK using less stringent regular expression patterns. There are two possible explanations for this phenomenon. On one hand, mitoK may be an additional regulatory factor for the potassium current regulated by ABCB8 and not the potassium channel itself. This seems unlikely, since the ABCB8/mitoK purified and reconstituted from bacteria exhibited potassium currents [56], and it is difficult to imagine this complex carrying a third protein (a potassium channel) during that complicated process. On the other hand, mitoK may represent a novel type of potassium channel, but AF-Multimer was not able to produce a meaningful prediction, since no previously known structure resembled this fold in its learning set. This is also unlikely in the light of our earlier study, demonstrating that AF2 was able to predict novel transmembrane folds not included in the learning and template sets [23].

### 2.4. The ABC3D Database

Since it is not trivial to access a list or a sublist of ABC proteins neither from the PDB nor from the AlphaFold Structural Database, we created a web application that contains the currently available full-length ABC structures (https://abc3d.hegelab.org). In this web application, structures can be browsed and searched in a categorized manner at various levels. On the first level, users can choose to display “Experimental”, “Computational”, or “Human” proteins and they can also view a comprehensive list of every available 3D structure under the “All” menu item. Every category except Human is further grouped by structural classes (Pgp-like, ABCG2-like, MalFG-like, EcfT-like, BtuC-like, LptFG-like, MacB-like, Bce-like, and MlaE-like) and conformations (open or closed). In the Human category, we chose to group proteins by subfamilies (ABCA, ABCB, ABCC, ABCG, and ABCD) instead of structural classes. Not only is this arrangement more common in the field, but also every human ABC protein can be classified into just two structural classes: ABCA and ABCG proteins belong to the ABCG2-like conformations, while ABCB, ABCC, and ABCD families exhibit Pgp-like structures.

One structural class or human subfamily can be selected by a single click, as well as structures with a specific conformation. Using the main Select button, more complex searches can be performed. Selections can be narrowed by not only structural class and conformation, but also by taxonomy, gene and protein names, and release dates. Each structure can also be selected or unselected individually. Three-dimensional structures can be visualized in the browser using Mol* [59] which helps the selection process. All structures are aligned to their corresponding reference structure which helps in comparison.

Selected structures and associated data can be downloaded as pdb files and a tab-separated tsv format, respectively. mmCif files are also available for bulk download. All of the files are packed into a zipped file. Users can also opt to download only the metadata file. The main Download menu makes getting pre-assembled files for each structural class possible.

### 2.5. Novel ABC Structures Accessible through the 3D-Beacons Network

In order to increase the visibility of our AF2-predicted ABC structures, we expose them through the 3D-Beacon Network (https://3d-beacons.org accessed on 20 June 2022). This network provides access to both theoretical and experimentally determined structures. In addition to providing programmatic access to our models, we also have web pages for each individual model (e.g., https://3dbeacon.hegelab.org/uniprot/Q9H222/hege-abc-0001 accessed on 20 June 2022). Our implementation of the 3D-Beacons Client has some additional fields which are not present in the current standard definitions (https://3dbeacons.docs.apiary.io/#, accessed on 6 June 2022) so that users can have access to the methodology, publication, and coordinate files other than the main mmCIF file. We found the latter important since some AlphaFold2 predictions contain structural regions which were most likely incorrectly built (e.g., soluble regions that enter the volume of the putative membrane region). In these cases, we published the trimmed version of the AF2-model as the main entry, but the full structure is also included on the webpage (e.g., ABCB10 homodimer, https://3dbeacon.hegelab.org/uniprot/Q9NRK6/hege-abc-0009, accessed on 20 June 2022). We also provide links to zip files containing all the structure files to download in different versions and formats easily.

## 3. Discussion

With the growing number of available structures in 3D databases, there is an increasing need for simple accession of several or all structures of a specific protein family. We developed a web application providing this search functionality for transmembrane ABC proteins and our approach can serve as a prototype for accessing bulk structural data per protein families. Based on user feedback, it will be improved and standardized to build a general framework for accessing the 3D structural data of any protein family. The focus of the further developments will be on the high-level automation of various steps (e.g., classification, structure collection, alignment). In this study, we automated the categorization of open/closed structures that was based on our manual curation of 325 experimental structures. This categorization has a limitation of hiding a finer grained clustering of structures reflecting also on occluded or various intermediate states. The automatic detection of these states requires challenging text and 3D data mining methods and will be addressed in future studies. However, the experimental conditions during structure determination may influence the conformationally flexible ABC protein structures [60,61], thus correct classification. It also remained unresolved for the static ABC structure sets, and how to address protein dynamics [60]. Extraction of protein motion using molecular dynamics simulations with the collected ABC structures is highly resource intensive [47,62]. Simplified approaches (e.g., normal mode analysis based on various network models [63,64]) may provide useful information on the dynamics of various conformers.

In addition to existing ABC structures, we exposed novel, AlphaFold-generated, dimeric structures of half ABC transporters through the implementation of the 3D-Beacons Client (https://3dbeacon.hegelab.org). In addition to current standard data fields, we found it important to attach extra information, such as methods and references to publications, which types of data are under heavy standardization by the 3D-Beacon community, extending the current fields.

Our results also highlighted some issues associated with the increasing number of available structures of proteins and protein complexes. First, characteristic conserved regions and structures should be identified more precisely than current approaches allow. Deep learning methods may allow domain identification with higher accuracy than an HMM profile search [65]. Second, high performance methods with novel and automatic logical processes are required to analyze and compare the structures of protein complexes efficiently (Figure S5). Our structure predictions strengthen our previous results [23] that AF models of TM proteins can be as high quality as that of soluble proteins. AlphaFold-Multimer also performs outstandingly for multimers, including TM ABC proteins. It provides higher performance for homomeric interfaces than for heteromeric interfaces which is presumably because MSA of homomeric proteins readily encodes evolutionary information about the complex interfaces, while this information for heteromeric interfaces is more limited and harder to access [36]. However, careful investigation of individual structures is required to help assess the model quality and also to detect hints for interesting and important biological questions, such as the case of the mitoSUR/mitoK complex (Figure 5). This example emphasizes various current limitations of AF ABC models. Besides the single chain predictions of otherwise multimeric proteins deposited to the AlphaFold Structural Database, the models do not involve ions, glycosylation, inhibitors, and substrate molecules that will be likely solved in the near future [66,67]. The predicted structures are also static and lack information on protein dynamics, except for regions with low pLDDT scores, indicating highly dynamic disordered parts [21]. However, the spatial information associated with these regions cannot be used in the case of many ABC proteins, since these regions possess pure predictive values and unrealistically cross the lipid bilayer region in some predicted structures [23]. In order to alleviate these issues for human ABC proteins, we made the set of human ABC protein structures complete with dimeric AlphaFold predictions, where unrealistic disordered regions were removed, which opens the way to merge and improve our ABC mutation database (http://abcm2.hegelab.org) [68,69] with this complete set of structures, thus interpreting any mutations of human ABC proteins in the context of their sequence and structure.

## 4. Methods and Materials

### 4.1. Databases and Associated Software

Experimental structures and associated sequences (release on 29 December 2021) were downloaded from RCSB. AlphaFold predicted structures of 21 proteomes were retrieved from the AlphaFold Structure Database in July 2021 [22]. These sets were used for analysis in this study, except for a few cases, which were raised after updating our database with current experimental structures and the AF structures covering proteins in SwissProt.

ABC PFAM entries were identified at https://pfam.xfam.org (accessed on 1 August 2021) (*n* = 29) and extracted from the Pfam-A.hmm file. The selected entries and their accession numbers are listed in our previous publication [23] and can be downloaded from http://abc3d.hegelab.org/pub/abcdoms.hmm (accessed on 5 July 2022). The sequence of every structure in the PDB and AF datasets was searched using HMMER hmmsearch 3.2.1 (June 2018), created by Howard Hughes Medical Institute, Maryland, US (http://hmmer.org) [70]. The E parameter was set to 0.001 and the match length was restricted to a minimum of 90% of the HMM profile length. The hmmsearch output was parsed using BioPython 1.79 [71].

### 4.2. Identification and Classification of TM ABC Proteins

Entries containing at least one TM Pfam hit and at least two NBD Pfam hits in the PDB and AF dataset sequences were selected and classified as transmembrane ABC proteins. Based on this comprehensive collection, corresponding biological unit files (.pdb1) and structural prediction files (.pdb) were downloaded from the PDB and AlphaFold Structural Database, respectively. The downloaded structures were then classified into structural families based on both Pfam hits and structural similarity to selected reference structures, listed in Table S1. From the sequence search results, PDB entries that included at least two NBDs and one TMD in the biological unit file (encoded in one or more chains) were selected as functional ABC protein structures. Then, TM-align was performed aligning every single structure to the reference structures, using the TM-score for characterizing structural similarity and to assure the reliability of the classification [72]. We considered two structures belonging to the same structural class if the TM-score resulting from their alignment was above 0.6, e.g., the Bce-like family emerged as a MacB Pfam hit with low structural similarity to the MacB reference structure (TM-score of 0.49).

To further classify the structures into inward-facing and inward-closed conformations, we used the distance between the Cα atom of the terminating amino acid of the Walker A helix (GXXXXG**K**T) in one of the NBDs and the signature helix (LS**G**GQ) in the other NBD. Since the signature sequence can be degenerate and cannot be recognized by sequence search, we identified these positions based on structural alignments with the Pgp NBD1 from 6c0v, using TM-align [72]. Structures were analyzed using MDAnalysis 2.0.0. [73] and NumPy 1.19.4. [74].

### 4.3. A Pipeline for Automatic Updates

In order to keep our collection updated, a Python script checks the sequence files deposited in PDBe and AlphaFold DB weekly. In case of changes, the aforementioned steps are executed automatically on the sequence of new entries. If the algorithm finds two NBD Pfam hits and no TMD hit in the sequences of the chains, we manually check the corresponding structure to avoid missing structures with novel transmembrane folds. Additional data needed for classification and the determination of conformational states is collected from cif files and UniProt [75]. The collected information is stored in a PostgreSQL database version 12.11 (https://www.postgresql.org). To make the appearance of structures on our website more uniform, the pdb files of identified ABC structures are aligned to the corresponding reference structure, regarding both structural family and conformational state, using the rmsd function of PyMOL (The PyMOL Molecular Graphics System, Version 2.4.0 Schrödinger, LLC, New York, NY, USA). The final pdb files were converted to mmCif files with gemmi [76].

### 4.4. Web Applications

Both the 3dbeacon and the abc3d web applications are based on FastAPI 0.71.0 (https://fastapi.tiangolo.com) placed behind an nginx 1.14.2 (https://www.nginx.com) web server. All data for our 3dbeacon client is stored in json files. The abc3d application data are tied to the PostgreSQL database via the SQLAlchemy object-relational mapper [77]. Web layout strongly depends on JavaScript, Jquery 3.6.0. (https://jquery.com), and bootstrap 5.1.3 (https://getbootstrap.com).

### 4.5. Running AlphaFold2

AlphaFold initial release and v2.0.0 were downloaded from github and installed as described (https://github.com/deepmind/alphafold) under Linux (Debian 10, 96 GB RAM, NVidia Quadro P6000 GPU with 24 GB RAM or NVidia RTX A6000 GPU with 48GB RAM GPU). We introduced minor modifications into the code to overcome memory usage problems in case of large multiple sequence alignment files and to be able to run multimer predictions with the initial release (http://alphafold.hegelab.org). Our runs used all genetic databases (*--db_preset=full_dbs*). Generated structures were evaluated based on PAE, pLDDT, and ipTM+pTM scores [21,36]. In addition, all top-scored structures were inspected visually.

### 4.6. Data Visualization

Molecular visualization was performed using PyMOL (The PyMOL Molecular Graphics System, Version 2.4.0 Schrödinger, LLC). Graphs were generated using Python 3.7.’s *matplotlib* 3.5.2. library [78].

## Data Availability

Web applications are available at https://3dbeacon.hegelab.org and https://abc3d.hegelab.org.

## Supplementary Materials

The supporting information can be downloaded at: https://www.mdpi.com/article/10.3390/ijms23168877/s1.
